# Impact of Immigration Status on Survival Among Stage 1 and 2 HER2‐Positive and Triple‐Negative Breast Cancer in Ontario, Canada

**DOI:** 10.1002/cam4.71288

**Published:** 2025-09-30

**Authors:** Omolara Fatiregun, Rinku Sutradhar, Sho Podolsky, Andrea Eisen, Lawrence Paszat, Eileen Rakovitch

**Affiliations:** ^1^ Institute for Health Policy, Management and Evaluation University of Toronto Toronto Ontario Canada; ^2^ Department of Radiology (Clinical Oncology) Lagos State University College of Medicine Lagos Nigeria; ^3^ Institute for Clinical Evaluative Sciences (ICES) Toronto Ontario Canada; ^4^ Division of Biostatistics, Dalla Lana School of Public Health University of Toronto Toronto Ontario Canada; ^5^ Sunnybrook Health Sciences Centre Sunnybrook Research Institute Toronto Ontario Canada; ^6^ Department of Medicine Sunnybrook Health Science Centre Toronto Ontario Canada; ^7^ Department of Radiation Oncology Sunnybrook Health Sciences Centre, Odette Cancer Centre Toronto Ontario Canada

**Keywords:** breast cancer, Her‐2 positive, immigration status, Ontario, triple negative

## Abstract

**Background:**

This study examined death from breast cancer and death from other causes among women with Stage 1 and 2 Her2‐positive and triple‐negative breast cancer (BC) by immigration status.

**Methods:**

We identified women aged 18–75 diagnosed with BC in Ontario from January 1, 2012, to December 31, 2019, followed them to December 31, 2023, and identified legal immigrants from the Immigration, Refugee, and Citizenship Canada Permanent Resident (CIC) database. We linked administrative data sources for the date of diagnosis, molecular subtype, death due to breast cancer, and death due to all other causes. Using adjusted competing risks regression (Fine and Gray method), we analyzed the influence of immigration on breast cancer survival and calculated the sub‐distribution hazard ratios (sHR).

**Results:**

There was no increased risk of death among legal immigrants on univariate or multivariable analysis. They had a sHR of 0.95 (0.77–1.19) on univariate analysis and 1.06 (95% CI: 0.83–1.36) on multivariable analysis for breast cancer deaths, and for other causes of death, 0.63 (0.47–0.83) on univariate analysis, and 0.85 (95% CI: 0.62–1.15) on multivariable analysis compared to long‐term residents. Patients with HER2‐positive status had a lower risk of death from breast cancer and other causes compared to those with triple‐negative breast cancer (TNBC). Patients with Stage 2 cancer had a significantly higher hazard of death from breast cancer compared to Stage 1 (HR = 3.72, 95% CI: 2.96–4.66, *p* < 0.0001).

**Conclusions:**

In Ontario, legal immigrants do not have an increased risk of death from breast cancer or other causes compared to long‐term residents.

## 1 Introduction

Breast cancer remains a significant global health challenge and a leading cause of morbidity and mortality among women worldwide, and understanding disparities in survival outcomes is critical for guiding public health interventions [1]. While advancements in screening and treatment have improved overall survival rates, HER2‐positive and triple‐negative BC (TNBC) remain two of the most aggressive subtypes associated with more aggressive disease progression and poorer prognosis, affecting women globally [2, 3, 4] Stage 1 and 2 HER2‐positive patients benefit from adjuvant chemotherapy plus targeted therapies, which significantly improve survival rates. Stage 1 and 2 TNBC patients also benefit from adjuvant chemotherapy. TNBC patients experience the lowest 5‐year overall survival rate (80.1%) compared to HER2+ cases, which fare better, with a rate over 90% [5]. Evaluating outcomes specifically for HER2+ and TNBC subtypes is important, as any potential disparities related to access to specialized treatments (e.g., timely anti‐HER2 therapy) or management of aggressive disease could disproportionately affect these groups.

Despite overall survival gains, disparities in breast cancer survival may persist across different population groups, influenced by factors including marginalization, race/ethnicity, and immigration status [6, 7]. Immigrant populations, representing a growing proportion of many Western societies, often may face unique challenges within healthcare systems. Previous research has demonstrated that immigrant populations may experience differences in cancer screening, diagnosis, and treatment processes, potentially leading to varied survival outcomes [8, 9, 10]. Additional factors that could serve as potential barriers to treatment access include barriers associated with language and cultural understanding. Navigating complex systems, and sometimes, the consequences of marginalization or precarious employment may also affect treatment access [11]. Conversely, the “healthy immigrant effect,” where immigrants initially demonstrate better health and lower mortality rates than the native‐born population, has also been widely observed, suggesting that immigrants often arrive healthier due to stringent health screenings and healthier lifestyles [12], which may potentially influence cancer survival outcomes [13, 14, 15]. Research examining cancer survival among immigrants has yielded mixed results; some studies report poorer outcomes attributed to later diagnosis or access barriers [11], while other reports, particularly within universal healthcare systems like Canada's, find comparable or even slightly better survival after adjusting for relevant factors [15]. Limited studies have examined the survival differences between immigrants and long‐term residents with HER2‐positive and TNBC in Ontario, employing a competing risks framework. This study evaluates the differences in breast cancer‐specific mortality and other‐cause mortality among immigrant and long‐term residents with Stage 1 and 2 HER2‐positive and triple‐negative breast cancer patients in Ontario.

## 2 Methods

### 2.1 Study Setting

Ontario is a province in Canada, with a population of over 14 million. The Ontario Health Insurance Plan (OHIP) provides healthcare benefits for all legal permanent residents.

### 2.2 Study Design

We conducted this population‐based retrospective cohort study by identifying women in the Ontario Cancer Registry between the ages of 18 and 75 diagnosed with breast cancer in Ontario from January 1st, 2012 to December 31st, 2019. We followed them up to December 31st, 2023, and stratified them into two groups: legal immigrants and long‐term residents, using the Immigration, Refugee, and Citizenship Canada Permanent Resident (CIC) database. Legal immigrants were defined as individuals who have been granted the right to live in Canada permanently, having moved from another country from 1986 or later, and therefore registered in the CIC database. Long‐term residents were defined as individuals already established in Canada and not included in the CIC database, including those who relocated between provinces in Canada. Using linked administrative data sources, we extracted information on the date of diagnosis, cancer stage, molecular subtype, demography, ACG System Resource Utilization Band (RUB), death due to breast cancer, and death due to all other causes. We included only Stage 1 and 2 breast cancer patients to ensure a homogenous study population of operable, early stage breast cancer potentially eligible for adjuvant or neoadjuvant chemotherapy. We excluded women with previous breast cancer in the OCR or a history of invasive non‐breast cancer malignancy within 10 years before the diagnosis, male patients, and women with pre‐invasive breast cancer (DCIS).

### 2.3 Data Sources

ICES is a non‐profit research institution in Canada that informs influential research and policy development by utilizing health and social data. Administrative datasets are linked using unique encoded identifiers and analyzed at the Institute for Clinical Evaluative Sciences (ICES). Data sources for this study include the Registered Persons Database (RPDB), Ontario Health Insurance Plan (OHIP), Citizenship and Immigration Canada IRCC Permanent Residents Database (CIC), Ontario Cancer Registry (OCR), Canadian Institute for Health Information Discharge Abstract Database (CIHI‐DAD), National Ambulatory Care Reporting System (CIHI‐NACRS) database, and the Same Day Surgery (CIHI‐SDS) database. Covariates included the following: Age at diagnosis was recorded from the OCR and categorized into three groups (< 50 years, 50–69 years, and > 70 years). We assessed marginalization using the ethnicity domain of the Ontario Marginalization Index (ONMARG). ONMARG is a census area‐based index used to show differences in marginalization and describe potential inequalities between geographical areas [16, 17, 18]. Each woman was assigned an ONMARG quintile based on her residence code.

To assess resource utilization and morbidity, we identified each woman's ACG System Resource Utilization Band (RUB) based on OHIP and CIHI records [19]. The RUB groups individuals using the same level of resources were assigned from one of six categories: 0—non‐user (lowest resource utilization), 1—healthy User, 2—low Morbidity, 3—moderate Morbidity, 4—high Morbidity, and 5—very High Morbidity (and resource utilization). Other covariates include estrogen receptor status, progesterone receptor status, and HER2 status.

### 2.4 Outcome Measures

The primary outcomes were breast cancer‐specific survival, defined as the time from the initial diagnosis to the date of death, specifically due to breast cancer, and the time to death from other causes. These events were treated as competing risks, as one type of death event precludes the occurrence of the other. Patients alive at the end of the study period or lost to follow‐up were censored at the date of last contact.

### 2.5 Statistical Analysis

We compared the baseline covariates of legal immigrants to long‐term residents and between patients who died during follow‐up and those who survived—summary statistics, including median survival times with interquartile ranges (IQR). Cumulative Incidence Functions (CIFS) were calculated and plotted to estimate the probability of death due to breast cancer and death due to other causes over time, accounting for the competing risks. To assess the association between legal immigration status and cause‐specific mortality while adjusting for potential confounders, multivariable competing risks regression models based on the Fine and Gray sub‐distribution hazard (SDH) approach were constructed [20]. Separate models were built for each outcome: death due to breast cancer (treating death from other causes as the competing event) and death from other causes (treating death due to breast cancer as the competing event). The Fine & Gray method was implemented to estimate adjusted sub‐distribution hazard ratios (sHR) with 95% confidence intervals (CI) for covariates, adjusting for age, molecular subtype, stage, resource utilization/morbidity, and demographic marginalization by ethnicity concentration quintile. We accounted for competing risks by censoring at the time of death due to a cause other than breast cancer, allowing for an unbiased estimation of the sub‐distribution hazard. All analyses used SAS version 9.4 (SAS Institute Inc., Cary, NC, USA). All statistical tests were two‐sided, 95% CL was estimated, and a *p*‐value of ≤ 0.05 was considered statistically significant.

## 3 Results

Table 1 presents the study population of the study cohort, comprising 8927 women, of whom 1617 (18.1%) were legal immigrants (540 with TNBC and 1077 with Her2‐positive disease), and 7310 (81.9%) were long‐term residents (2724 with TNBC and 4586 with Her2‐positive disease), across various baseline variables. The mean age of the total sample was 55.75 years (SD = 10.81). The mean age was significantly lower for legal immigrants (51.77 years, SD = 10.15) compared to long‐term residents (56.63 years, SD = 10.75), *p* < 0.0001. A higher percentage of legal immigrants had Stage 2 breast cancer (58.9%) compared to long‐term residents (52.6%), *p* < 0.0001. HER2‐positive receptor status was more prevalent among legal immigrants (66.6%) than among long‐term residents (62.7%), *p* = 0.004. Most legal immigrants (61.7%) were in the 5th quintile of the ONMARG ethnic quintile (highest marginalization), while long‐term residents were relatively uniformly distributed across all quintiles, *p* < 0.0001. Most women in both groups were classified in the RUB3 category (Immigrants, 60.8%; Long‐term residents, 61%).

**TABLE 1:** Study population: Stage 1 and 2 Her‐2 positive and triple‐negative breast cancer by immigration status and vital status.

Variable	Total	Immigrants (in CIC)	Long‐term residents (not in CIC)	*p*	Death—no	Death—yes	*p*
	*N* = 8927	*N* = 1617	*N* = 7310		*N* = 7895	*N* = 1032	
**Cancer stage, *n* (%)**							
Stage 1	4129 (46.3)	664 (41.1)	3465 (47.4)	< 0.0001	3851 (48.8)	278 (26.9)	< 0.0001
Stage 2	4798 (53.7)	953 (58.9)	3845 (52.6)		4044 (51.2)	754 (73.1)	
**Age (numeric)**							
Mean (SD)	55.75 (10.81)	51.77 (10.15)	56.63 (10.75)	< 0.0001	55.35 (10.68)	58.79 (11.28)	< 0.0001
Median (Q1–Q3)	56 (48–65)	51 (44–59)	57 (50–65)	< 0.0001	56 (48–64)	61 (51–69)	< 0.0001
**Age (categorical), *n* (%)**							
< 50	2525 (28.3)	701 (43.4)	1824 (25.0)	< 0.0001	2314 (29.3)	211 (20.4)	< 0.0001
50–69	5416 (60.7)	824 (51.0)	4592 (62.8)		4806 (60.9)	610 (59.1)	
≥ 70	986 (11.0)	92 (5.7)	894 (12.2)		775 (9.8)	211 (20.4)	
**Molecular subtype, *n* (%)**							
Triple negative	3264 (36.6)	540 (33.4)	2724 (37.3)	0.004	2723 (34.5)	541 (52.4)	< 0.0001
Her2 positive	5663 (63.4)	1077 (66.6)	4586 (62.7)		5172 (65.5)	491 (47.6)	
**ONMARG Ethnic Quintile**							
1	1577 (17.7)	44 (2.7)	1533 (21.0)	< 0.0001	1369 (17.3)	208 (20.2)	0.001
2	1603 (18.0)	69 (4.3)	1534 (21.0)		1388 (17.6)	215 (20.8)	
3	1696 (19.0)	128 (7.9)	1568 (21.5)		1496 (18.9)	200 (19.4)	
4	1890 (21.2)	379 (23.4)	1511 (20.7)		1709 (21.6)	181 (17.5)	
5	2161 (24.2)	997 (61.7)	1164 (15.9)		1933 (24.5)	228 (22.1)	
**Resource utilization band (RUB), *n* (%)**							
0–2	862 (9.7)	99 (6.1)	763 (10.4)	< 0.0001	792 (10.0)	70 (6.8)	< 0.0001
3	5444 (61.0)	983 (60.8)	4461 (61.0)		4883 (61.8)	561 (54.4)	
4	1893 (21.2)	415 (25.7)	1478 (20.2)		1647 (20.9)	246 (23.8)	
5	728 (8.2)	120 (7.4)	608 (8.3)		573 (7.3)	155 (15.0)	
**Length of follow‐up (in years)**							
Mean (SD)	5.28 (2.49)	5.25 (2.44)	5.29 (2.50)	0.53	5.40 (2.47)	4.34 (2.43)	< 0.0001
Median (Q1–Q3)	5 (4–7)	5 (4–7)	5 (4–7)	0.66	5 (4–7)	4 (2–6)	< 0.0001

**FIGURE 1:**
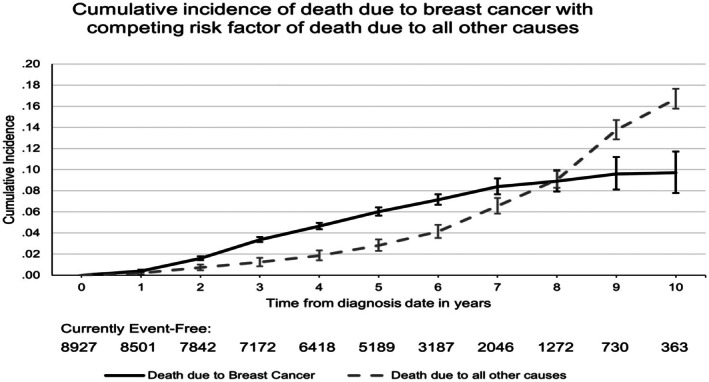
The cumulative incidence of death due to breast cancer and other causes.

**FIGURE 2:**
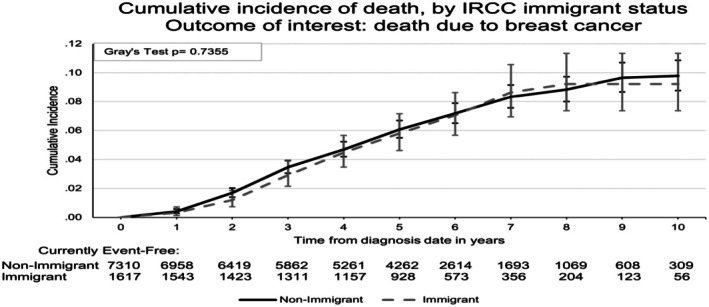
The cumulative incidence of death by immigration status (legal immigrant vs. long‐term resident) for breast cancer.

**FIGURE 3:**
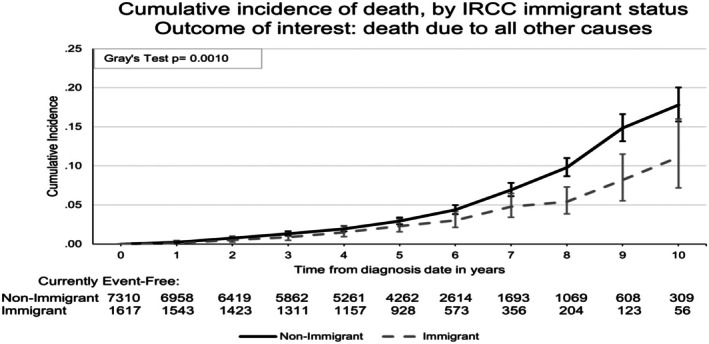
The cumulative incidence of death by immigration status for other causes.

Table 1 also shows the vital status at the last follow‐up of Stage 1 and 2 HER2‐positive or triple‐negative breast cancer patients. The proportion of legal immigrants who died during follow‐up was lower (9.5% vs. 12.0%), *p* = 0.004. A total of 1032 patients (11.6%) died, while 7895 (88.4%) survived. Among those who died, only 26.9% were Stage 1, while 73.1% were Stage 2 (*p* < 0.0001). More patients with TNBC, 52.4%, died due to breast cancer, compared to 47.6% among Her2‐positive patients (*p* < 0.0001). The resource utilization/morbidity (RUB) distribution also differed significantly, with a higher percentage of deceased patients in the RUB Categories 4 and 5. The cumulative incidence of death due to breast cancer and death due to all other causes increases steadily over time (Figure 1) The cumulative incidence of death from breast cancer is not significantly different between legal immigrants and long‐term residents (Gray's test *p* = 0.74) (Figure 2). Legal immigrants exhibited a statistically lower incidence of death due to other causes (Gray's test *p* = 0.0010; Figure 3).

In Table 2, there was no increased risk of death among legal immigrants at both univariate and multivariable analysis. They had a sub‐distribution Hazard Ratio [sHR], 0.95, 95% Confidence Interval [CI]: 0.77–1.19, on univariate analysis and sHR, 1.06 (95% CI: 0.83–1.36) on multivariable analysis for breast cancer deaths, and for other causes of death, sHR 0.63 (95% CI: 0.47–0.83) on univariate analysis, and sHR 0.85 (95% CI: 0.62–1.15) on multivariable analysis compared to long‐term residents. Her2‐positive status was associated with a significantly lower hazard of death from breast cancer (HR = 0.35, 95% CI: 0.30–0.42, *p* < 0.0001) but was not significantly associated with death from other causes (*p* = 0.29). Patients with Stage 2 cancer had a significantly higher hazard of death from breast cancer compared to Stage 1 (HR = 3.72, 95% CI: 2.96–4.66, *p* < 0.0001). Stage 2 was also associated with a significantly higher hazard of death from other causes, though the effect size was smaller (HR = 1.23, 95% CI: 1.02–1.48, *p* = 0.03). Compared to the reference group (0–2), RUB level 5 was associated with a significantly increased hazard of death from breast cancer (HR = 1.62, 95% CI: 1.11–2.38, *p* = 0.01). For death from other causes, RUB levels 4 (HR = 2.02, 95% CI: 1.32–3.09, *p* = 0.001) and 5 (HR = 2.99, 95% CI: 1.93–4.65, *p* < 0.0001) showed significantly increased hazards compared to the reference group. None of the ONMARG demographic ethnicity concentration quintiles showed a significant association with death from breast cancer (all *p* > 0.05). For death from other causes, the ONMARG Ethnic Q Category 5 was associated with a significantly lower hazard (HR = 0.72, 95% CI: 0.54–0.98, *p* = 0.005). Increasing age was significantly associated with a higher hazard for both outcomes. Compared to patients < 50 years old, those aged 50–69 and 70+ had significantly higher hazards of death from breast cancer (HR = 1.30, *p* = 0.01 and HR = 2.25, *p* < 0.0001, respectively). The hazards increase with age were even more pronounced for death from other causes (Age 50–69: HR = 2.21, *p* < 0.0001; Age 70+: HR = 5.50, *p* < 0.0001).

**TABLE 2:** Sub‐distribution hazard ratios for death due to breast cancer and other causes | Stages 1 and 2.

Variables	Outcome of interest: death due to breast cancer competing event: death due to other causes		Outcome of interest: death due to other causes competing event: death due to breast cancer	
Parameter	Univariate SHR (95% CI)	Multivariate SHR (95% CI)	Univariate SHR (95% CI)	Multivariate SHR (95% CI)
**In CIC‐IRCC**				
No	Ref	Ref	Ref	Ref
Yes	0.95 (0.77–1.19)	1.06 (0.83–1.36)	0.63 (0.47–0.83)	0.85 (0.62–1.15)
Triple negative	Ref	Ref	Ref	Ref
Her2	0.32 (0.27–0.38)	0.35 (0.30–0.42)	0.84 (0.70–1.00)	0.83 (0.69–0.99)
**Cancer stage**				
Stage 1	Ref	Ref	Ref	Ref
Stage 2	3.44 (2.75–4.29)	3.75 (2.99–4.69)	1.12 (0.93–1.34)	1.36 (1.13–1.64)
**RUB**				
0–2	Ref	Ref	Ref	Ref
3	1.15 (0.84–1.57)	1.06 (0.77–1.45)	1.36 (0.92–2.01)	1.41 (0.94–2.12)
4	1.30 (0.92–1.83)	1.21 (0.86–1.72)	1.97 (1.31–2.97)	2.07 (1.35–3.17)
5	1.85 (1.27–2.70)	1.70 (1.17–2.49)	3.50 (2.8–5.37)	3.13 (2.01–4.88)
**ONMARG Ethnic Q**				
1	Ref	Ref	Ref	Ref
2	1.11 (0.85–1.45)	1.07 (0.82–1.39)	0.81 (0.62–1.07)	0.83 (0.63–1.09)
3	0.82 (0.62–1.08)	0.87 (0.66–1.15)	0.87 (0.67–1.14)	0.93 (0.71–1.21)
4	0.84 (0.64–1.11)	0.87 (0.66–1.14)	0.58 (0.43–0.78)	0.64 (0.48–0.87)
5	0.91 (0.71–1.18)	0.93 (0.70–1.23)	0.60 (0.45–0.79)	0.72 (0.54–0.98)
**Age**				
< 50	Ref	Ref	Ref	Ref
50–69	1.12 (0.92–1.36)	1.30 (1.06–1.59)	2.37 (1.82–3.10)	2.20 (1.68–2.89)
> 70	1.85 (1.43–2.41)	2.25 (1.70–2.97)	6.45 (4.79–8.68)	5.50 (4.05–7.45)

Abbreviations: 95% CI, 95% Confidence Interval; Ethnic Q, Ethnic Quintile; RUB, Resource Utilization Band in CIC‐IRCC (legal immigrant = Yes, Long‐term resident = No); SHR, Sub‐distribution Hazard Ratio.

## 4 Discussion

This study compares survival outcomes among patients diagnosed with Stage 1 and 2 Her2‐positive and TNBC between legal immigrants and long‐term residents, utilizing a competing risks framework to distinguish between death due to breast cancer and death from other causes while adjusting for key demographic and clinical covariates. Our primary finding shows that legal immigrants did not have an increased hazard of death due to either breast cancer‐specific mortality or mortality from other causes after accounting for factors such as Her2 status, cancer stage, RUB score, demographic ethnicity concentration quintile, and age. There was also no increased risk of death among immigrants at either univariate or multivariable analyses.

Our analysis confirmed several known prognostic factors consistent with established breast cancer literature [1, 21]. HER2‐positive status was significantly protective against breast cancer death compared to triple‐negative status. Interestingly, the proportion of HER2‐positive breast cancer was higher among legal immigrants than long‐term residents. HER2‐positive breast cancer patients have seen significantly improved survival rates compared to those with TNBC, primarily due to the inclusion of effective anti‐HER2 treatments in adjuvant therapy [2, 22]. As expected, Stage 2 disease carried a significantly higher risk of both breast cancer‐specific death and other‐cause mortality compared to Stage 1 [23]. Increasing age was strongly associated with poorer outcomes for both endpoints, but markedly more so for death from other causes than for breast cancer death. This finding is consistent with prior research, which identifies advanced age as a common predictor of poorer outcomes due to several factors [24, 25]. This highlights the critical importance of using a competing risks framework, as older patients are substantially more likely to die from non‐cancer causes, and standard survival analyses could misattribute this risk [26, 27]. These trends highlight the necessity for age‐specific strategies in breast cancer management, including enhanced screening and tailored therapeutic approaches.

Higher resource utilization/morbidity (RUB) scores (Levels 4 and 5) were also associated with increased mortality, particularly from other causes, reflecting higher comorbidity burdens or frailty not fully captured by age alone. This suggests a complex relationship between morbidity and patient survival, which aligns with existing literature on the relationship between healthcare utilization and patient survival rates. For instance, studies have shown that patients with greater healthcare utilization often have more complex health needs, which can complicate treatment and lead to poorer survival outcomes [28].

## 5 Strengths

A key strength of this study is the application of a competing risks analysis of mortality patterns in this population. Understanding survival outcomes in breast cancer requires understanding the competing risks of other causes of death events that preclude the occurrence of the primary event of interest, death from breast cancer [29]. Survival analyses, such as Kaplan–Meier and Cox proportional hazards models, censor observations at the occurrence of these competing risks, potentially leading to biased estimates of survival probabilities and hazard ratios. Consequently, competing risk analysis is an important methodological framework, especially in settings with considerable heterogeneity in patient backgrounds and health outcomes [26, 29, 30]. The adjustment for multiple important clinical and demographic covariates also strengthens our findings regarding the independent effect of being a legal immigrant compared to a long‐term resident. Overall, the study highlights critical factors such as age, cancer stage, marginalization, and resource utilization that might influence survival in Stages 1 and 2 breast cancer patients with HER2+ and triple‐negative subtypes. By employing a competing risks approach, this study provides more precise and nuanced insights into the survival dynamics of breast cancer patients.

## 6 Limitations

While this study contributes valuable insights, limitations should be acknowledged. This research relies on administrative databases and retrospective data, which may lead to biases in patient selection and treatment categorization, as well as potential misclassification and missing data [31]. Additionally, these findings are specific to patients with Stage 1 and 2 breast cancer within the studied healthcare system and may not be generalizable to other populations, cancer stages, or settings. The ICES datasets contain only individuals eligible for the Ontario Health Insurance Plan (OHIP) coverage; as a result, we have no records regarding individuals without coverage and immigrants without legal status. Our findings, therefore, are not applicable to them. The CIC database does not account for immigrants who landed in other provinces and then moved to Ontario. We employed the ethnicity concentration domain of the Ontario Marginalization Index (ONMARG) to adjust for marginalization associated with racialization among both legal immigrants and long‐term residents. ONMARG Index is an area‐based measure of marginalization, rather than based on characteristics and experiences of an individual woman [32].

## 7 Conclusion

In this cohort of Stage 1 and 2 HER‐2 positive and triple‐negative breast cancer patients, being a legal immigrant was not associated with an increased risk of death from either breast cancer‐specific mortality or death from other causes on univariate or multivariable analysis compared to long‐term residents.

## Author Contributions

**Omolara Fatiregun:** conceptualization, investigation, writing – original draft, methodology, validation, visualization, writing – review and editing, formal analysis, data curation. **Rinku Sutradhar:** conceptualization, methodology, supervision, formal analysis. **Sho Podolsky:** methodology, formal analysis, visualization. **Andrea Eisen:** conceptualization, methodology, writing – review and editing, supervision, validation. **Lawrence Paszat:** conceptualization, writing – original draft, investigation, methodology, visualization, writing – review and editing, formal analysis, data curation, supervision. **Eileen Rakovitch:** conceptualization, methodology, investigation, writing – original draft, writing – review and editing, supervision, formal analysis.

## Conflicts of Interest

The authors declare no conflicts of interest.

## Data Availability

The data that support the findings of this study are available from the Institute for Clinical Evaluative Sciences, but restrictions apply to the availability of these data, which were used under license for the current study and so are not publicly available.
